# Essential oils: in vitro activity against *Leishmania amazonensis*, cytotoxicity and chemical composition

**DOI:** 10.1186/s12906-016-1401-9

**Published:** 2016-11-08

**Authors:** Milene Aparecida Andrade, Clênia dos Santos Azevedo, Flávia Nader Motta, Maria Lucília dos Santos, Camila Lasse Silva, Jaime Martins de Santana, Izabela M. D. Bastos

**Affiliations:** 1 Pathogen-Host Interface Laboratory, Department of Cell Biology, The University of Brasília, Campus Darcy Ribeiro, Bloco I, Brasília, DF CEP 70910-900 Brazil; 2 Faculty of Ceilândia, The University of Brasília, Brasília, Brazil; 3 Institute of Chemistry, The University of Brasilia, Brasília, Brazil

**Keywords:** Secondary metabolites, Anti-*Leishmania*, Natural products, *Ferula galbaniflua*

## Abstract

**Background:**

The current chemotherapy for cutaneous leishmaniosis (CL) has a series of drug limitations such as toxic side effects, long duration, high costs and drug resistance, which requires the development of new drugs or effective alternatives to the CL treatment. Essential oils (EOs) are complex mixtures of secondary metabolites from various plants. It has been shown that several EOs, or their constituents, have inhibitory activity against protozoa. Thus, this study aims to evaluate the biological activity of different essential oils (EOs) on *Leishmania* (*L*.) *amazonensis* promastigotes forms, as well as their cytotoxicity on mammalian cells and chemical composition.

**Methods:**

Sixteen EOs were evaluated by mean of IC_50_/24 h and cytotoxicity against L6 cells (CC_50_/24 h) using Resazurin assay. Only those EOs that presented better results for IC_50_/24 h were submitted to GC–MS analysis to determine their chemical constitution.

**Results:**

The EO from *Cinnamodendron dinisii*, *Matricaria chamomilla*, *Myroxylon peruiferum*, *Salvia sclarea*, *Bulnesia sarmientoi*, *Ferula galbaniflua*, *Siparuna guianensis* and *Melissa officinalis* were the most active against *L. amazonensis* with IC50/24 h ranging from 54.05 to 162.25 μg/mL. Analysis of EOs by GC–MS showed mainly the presence of β-farnesene (52.73 %) and bisabolol oxide (12.09 %) for *M. chamomilla*; α-copaene (13.41 %), safrole (8.35 %) and δ-cadinene (7.08 %) for *M. peruiferum*; linalool (28.80 %) and linalyl acetate (60.08 %) for *S. sclarea*; guaiol (48.29 %) and 2-undecanone (19.49 %) for *B. sarmientoi*; ethyl phthalate (13.09 %) and methyl-8-pimaren-18-oate (41.82 %) for *F. galbaniflua*; and neral (37.18 %) and citral (5.02 %) for *M. officinalis*.

**Conclusion:**

The EO from *F. galbaniflua* showed to be effective against *L. amazonensis* promastigotes forms and presented low cytotoxic activity against L6 cells. Thus, it represents a strong candidate for future studies aiming its molecular activity on these pathogenic parasites.

## Background

Leishmaniasis, the third most important vector-borne diseases, is caused by a protozoan parasite of the genus *Leishmania*, which is transmitted to human by the bite of sand flies. Leishmaniasis represents a complex disease with diverse clinical manifestations and poses a public health problem since it is a neglected tropical disease with current high worldwide incidence [1, 2]. Globally, more than 12 million individuals are infected, with another 350 million at risk of infection, and nearly 2 million new cases are reported annually worldwide [3]. The disease is prevalent in 16 developed and 72 developing countries; nevertheless 90 % of cases are reported in three regions: Sudan/Ethiopia/Kenya, India/Bangladesh/Nepal and Brazil with as many as 0.02 to 0.04 million deaths every year [3, 4].

Leishmaniasis can be divided into three forms, varying in severity from self-healing cutaneous lesions, dermatological ulcers in cutaneous leishmaniasis (CL), destructive form of mucocutaneous leishmaniasis, to deadly form of visceral leishmaniasis (VL) [5]. CL is characterized by ulcers on the skin that are often formed at the site of the insect vector bite. Those ulcers can undergo metastasis of the nasopharyngeal mucosa developing to tissue destruction, depending on the species of *Leishmania* involved [6]. *Leishmania (Viannia) braziliensis* and *Leishmania (V.) panamensis* are responsible for cases of mucocutaneous leishmaniasis in the Americas, although *L. (V.) guyanensis* and *L. (L.) amazonensis* have been identified, especially, in immuno-compromised hosts [6].

The first-line drugs for systemic treatment of leishmaniasis are parenterally administered antimonials such as the sodium stibogluconate (Pentostam®) and the N-methyl glucamine antimoniate (Glucantime®) [7, 8] generally required for the treatment of CL in the New World due to the risk of mucosal involvement [9]. This current chemotherapy presents several issues such as high cost, difficult administration and elevated toxicity, associated with serious side effects [10], for instance musculoskeletal pain, gastrointestinal disturbances, mild to moderate headache, electrocardiographic QTs interval prolongation and mild to moderate increase of liver and pancreatic enzymes [11]. Second-line drug Pentamidine and amphotericin B are not widely used due to their high toxicity and cost. Miltefosine, the first oral anti-leishmanial drug, is the treatment of choice for diffuse cutaneous leishmaniasis and New World cutaneous leishmaniasis caused by *Leishmania braziliensis* but increasing resistance to this drug has been notified [12].

All antileishmanial drugs except miltefosine have to be administrated parenterally. Most of these drugs are toxic, requires prolonged hospitalization and close monitoring, which makes the treatment costly and beyond the reach of most patients. Consequently, the development of alternative therapies is a priority for the treatment of leishmaniasis. As a strategy, the investigation of extracts and compounds, with biological activity, isolated from plants and used in traditional medicine is a promising in the research field for compounds with potential action for the prophylaxis and chemotherapy of CL [13].

Essential oils (EOs) are complex mixtures of secondary metabolites isolated from plants. In these mixtures, there are 10–60 constituents at different concentrations, but usually only 2–3 major constituents determine the biological properties of the EO [14]. Those compounds and their constituents present a broad pharmacological spectrum, and they are used as analgesics, sedatives, anti-inflammatory, and anti-spasmodic drugs, as well as antimicrobials, antiprotozoals and antihelmintics [13, 15, 16]. It has been shown that several EOs or their constituents have inhibitory activity on protozoa, especially *Leishmania* [17–19]. For instance, Santos and colleagues demonstrated that copaiba oil from *Copaifera martii* is a safer, shorter, less-expansive, and more easily administered antileishmanial drug [18]. Therefore, the purpose of this present work was to analyze the effect of sixteen EOs biological potential on *L. amazonensis* promastigotes forms and L6 cells and chemical constitution, by GC-MS, of those EOs that showed better leishmanicidal results.

## Methods

### Essential oils

EOs of *Litsea cubeba* fruits; *Lavandula officinalis*, *Matricaria chamomilla* and *Cananga odorata* flowers; *Elettaria cardamomum* seeds; *Cinnamomum camphora*, *Myroxylon peruiferum* and *Bulnesia sarmientoi* barks; *Ferula galbaniflua* resin; *Salvia sclarea*, *Foeniculum officinalis*, *Cordia verbenaceae*, and *Melissa officinalis* leaves; *Pelargonium graveolens* leaves and stems were purchased from QUINARI Cosmetic and Fragrances Inc. (Maringá-PR, Brazil) with lot number 0717/05209/F. EOs of *Cinnamodendron dinisii* and *Siparuna guianensis* leaves were obtained as described by Andrade et al. [20].

Firstly, EOs and Amphotericin B 250 μg/mL (Sigma-Aldrich, St. Louis, USA) were diluted in dimethylsulfoxide (DMSO) at 100 mg/mL and 50 μg/mL, respectively. For use, the stock was diluted 5:100 in either Schneider (Sigma-Aldrich) or RPMI-1640 (Sigma-Aldrich) media (sub stock). DMSO final concentration in the experiments never exceeded 0.5 %, a concentration that is not harmful to parasites and L6 cells [13]. Stocks were stored at 4 °C in the dark, to avoid degradation [21]. The sub stock was freshly prepared before use.

### Chemical composition of EOs

Gas chromatography–mass spectrometry (GC–MS) analysis was performed using a Shimadzu GC-2010 gas chromatograph coupled with GCMS-QP2010 Plus equipped with auto sampler (model AOC-20i, Shimadzu, Columbia, MD, USA) and GC–MS Solution software. Investigation was performed with a Rtx-5MS capillary column (30 mm × 0.25 mm × 0.25 μm) at programmed temperature ranging from 60 to 250 °C at 3 °C/min. Analysis conditions were: injector temperature 250 °C, ion source interface temperature 300 °C, analysis of masses between 40–350 m/z, electron impact at 70 eV, column head pressure at constant pressure of 59 kPa, column flow 1.02 mL/min, gas linear velocity: 36.8 cm/s, carrier gas: helium, injected volume 1 μL (1:1000 in hexane) in splitless. Constituents of EOs were identified by comparing their mass spectral pattern and retention indexes (RI) relative to a standard n-alkane series (C_9_–C_24_) with those known in the literature and the Wiley W9N08 database [13, 22].

### Parasites and culture conditions

The promastigotes forms of *L. amazonensis* (strain MHOM/BR/77/LTB0016) were maintained at 28 °C in Schneider medium supplemented with 10 % fetal bovine serum (FBS) and 100 μg/mL gentamicin, with weekly passages. For the screening of EOs biological potential, promastigotes were collected from cultures at the mid-log phase of growth (3-day-old culture). The parasite strain was obtained from Fiocruz-COLPROT (Coleção de Protozoários da Fiocruz).

### Antileishmanial activity of essential oils *in vitro*

EOs serial dilutions, from 500 to 31.25 μg/mL, were prepared on a 96-well cell culture plate. Afterwards, 1.35 × 10^6^ parasite/mL culture resuspended in 150 μL were added to the plates and incubated for 24 h at 28 °C. After this period, 20 μL of Resazurin solution were added to a 2 mM final concentration in all wells [23, 24]. The plates were incubated for further 4 h at 37 °C followed by the fluorescence measurement under 570 nm_ex_/595 nm_em_ in the microplate reader SpectraMax M5 (Molecular Devices, Sunnyvale, CA, USA).

*In vitro* experiment was performed in triplicate and repeated twice independently. DMSO was used as control in the same final concentration found in each dilution. Amphotericin B was used as positive control at final concentration ranging from 312.5 to 19.56 ng/mL.

The percentage of viable promastigotes cells was determined by the equation [25]:

$$ \%\mathrm{P}=\left(100\times \mathrm{F}\mathrm{a}\right)/\mathrm{F}\mathrm{c} $$

Where %P: percentage of viable promastigotes cells; Fc: control fluorescence units; Fa: fluorescence units emitted by the analyzed samples (with inhibitor).

### Cytotoxic activity of essential oils *in vitro*

Uninfected L6 cell monolayers were washed with Phosphate Buffered Saline (PBS) for 5 min at 37 °C, washed with RPMI medium pH 7.4 + 2.5 % FBS, centrifuged at 200 *g* for 10 min at 4 °C, resuspended in the same medium and, finally, seeded into 96-well plates (5 × 10^4^ cells/well). Plates were incubated at 37 °C for 24 h, then the medium was removed and cells were washed with PBS. Diluted EOs were added to overnight-adhered L6 cells and incubated for more 24 h at 37 °C. Cell viability was assessed by 2 mM Resazurin as described above.

The percentage of viable cells was determined by the equation [25]:

$$ \%\mathrm{V}=\left(100\times \mathrm{F}\mathrm{a}\right)/\mathrm{F}\mathrm{c} $$

Where %V is the percentage of viable cells, Fc: control fluorescence units; Fa: fluorescence units emitted by the analyzed samples (with inhibitor). The selectivity index (SI) was calculated by dividing CC_50_ for the IC_50_.

### Statistical analysis

For both *in vitro* EOs biological potential, a randomized complete block design (RBD) test was used, with 5 concentrations, 3 repetitions and 2 experiments (blocks) for each sample. The statistical program used was SISVAR [26]. Data were submitted to analysis of variance and the averages compared by Scott-Knott test and regression, both 5 % probability. The adjusted equations were used to calculate the concentration needed to cripple 50 % of L6 cells (CC_50_) or 50 % of the parasites (IC_50_).

## Results

### Antileishmanial and cytotoxic activity of essential oils *in vitro*

Growth inhibitory activity by the selected EOs was performed on *L. amazonensis* promastigotes forms at concentrations ranging from 30 to 500 μg/mL. In the test, the EOs of *L. cubeba*, *E. cardamomum*, *L. officinalis*, *C. camphora* and *C. odorata* did not show activity at 500 μg/mL (Table 1). Lower concentrations of the remaining EOs were then evaluated to estimate the IC_50_/24 h (Table 1). The most effective EO was of the one from *S. guianensis* (48.55 ± 3.64 μg/mL), followed by *C. dinisii* (54.05 ± 4.88 μg/mL), *M. chamomilla* (60.16 ± 4.24 μg/mL), *C. verbenaceae (*64.75 ± 2.04 μg/mL), *B. sarmientoi* (85.56 ± 3.38 μg/mL)*, F. galbaniflua* (95.70 ± 1.82 μg/mL), *M. officinalis* (132.02 ± 3.14 μg/mL)*, M. peruiferum* (162.25 ± 1.57 μg/mL)*, S. sclarea (*325.92 ± 8.58 μg/mL), *F. officinalis (*328, 28 ± 6,80 μg/mL) and *P. graveolens* (363.71 ± 6.77 μg/mL). The IC_50_/24 h of Amphotericin B was 0.83 ± 0.03 μg/mL (Table 1).

**Table 1:** EOs biological potential and selectivity indexes (SI) for *L. amazonensis* (IC_50_/24 h) promastigotes and L6 cells (CC_50_/24 h)

Essential oils	*L. amazonensis*	L6 cells	SI^g^
	IC_50_^a^ ± DP (μg/mL)	CC_50_^f^ ± DP (μg/mL)	
*Litsea cubeba*	NI^e^	180.72 ± 1.37	-
*Matricaria chamomilla*	60.16 ± 4.24	173.04 ± 1.24	2.87
*Elettaria cardamomum*	>500.00^b^	439.57 ± 2.27	–
*Lavandula officinalis*	>500.00	377.56 ± 8.91	–
*Cinnamomum camphora*	>500.00	>500.00	–
*Myroxylon peruiferum*	162.25 ± 1.57	160.80 ± 1.62	0.99
*Salvia sclarea*	325.92 ± 8.58	375.37 ± 3.62	1.15
*Bulnesia sarmientoi*	85.56 ± 3.38	163.46 ± 1.77	1.91
*Ferula galbaniflua*	95.70 ± 1.82	377.26 ± 2.71	3.94
*Pelargonium graveolens*	363.71 ± 6.77	368.39 ± 3.90	1.01
*Cananga odorata*	NI	142.80 ± 1.76	–
*Foeniculum officinalis*	328. 28 ± 6.80	368.27 ± 3.81	1.12
*Cordia verbenaceae*	64.75 ± 2.04	130.00 ± 1.08	2.01
*Melissa officinalis*	132.02 ± 3.14	297.45 ± 1.32	2.25
*Siparuna guianensis*	48.55 ± 3.64	78.02 ± 1.19	1.60
*Cinnamodendron dinisii*	54.05 ± 4.88	106.31 ± 2.23	1.97
Anfotericina B^c^	(0.083 ± 0.003 μg/mL)	NI	–
DMSO^d^	NI	NI	–

^a)^ IC_50_ ± DP: the concentration able to cripple 50 % of the parasites ± standard deviation

^b)^ > 500.00: IC_50_ greater than the highest concentration tested

^c)^ Amphotericin B - positive control

^d)^ DMSO - negative control

^e)^ NI: no inhibition

^f)^ CC_50_: the concentration able to cripple 50 % of cells after 24 h of treatment ± standard deviation

^g)^ Selectivity index - SI = CC_50_ L6 / IC_50_ promastigotes

The cytotoxicity against L6 cells and *L. amazonensis* were compared using the selectivity index (SI) (Table 1). Higher values of SI means more promising compounds for developing antileishmanial drugs. The SI measures the compound’s level of selectivity towards *L. amazonensis*. Evaluation of cytotoxicity showed that the least cytotoxic EO was that of *C. camphora* (CC_50_/24 h = >˃500.00 μg/mL), followed by *E. cardamomum* (439.57 ± 2.27 μg/mL), *L. officinalis* (377.56 ± 8.91 μg/mL), *F. galbaniflua* (377.26 ± 2.71 μg/mL), *S. sclarea* (375.37 ± 3.62 μg/mL), *P. graveolens* (368.39 ± 3.90 μg/mL), *F. officinalis* (368.27 ± 3.81 μg/mL), *M. officinalis* (297.45 ± 1.32 μg/mL), *L. cubeba* (180.72 ± 1.37 μg/mL), *M. chamomilla* (173.04 ± 1.24 μg/mL), *B. sarmientoi* (163.46 ± 1.77 μg/mL), *M. peruiferum* (160.80 ± 1.62 μg/mL), *C. odorata* (142.80 ± 1.76 μg/mL), *C. verbenaceae* (130.00 ± 1.08 1.77 μg/mL), *C. dinisii* (106.31 ± 2.23 μg/mL) and the most cytotoxic EO was of the one from *S. guianensis* (78.02 ± 1.19 μg/mL) (Table 1).

EOs with higher selectivity indexes were those from *F. galbaniflua* (3.94), *M. chamomilla* (2.87) and *M. officinalis* (2.25), but all were more cytotoxic and less selective than Amphotericin B, because the reference drug did not show CC_50_/24 h value.

### Chemical composition

The GC-MS analyses were performed for EOs that showed the lower values of IC_50_ and/or the higher SI values (Table 2). The analysis identified the main constituents as β-farnesene (52.73 %), bisabolol oxide (12.09 %), α-farnesene (10.34 %) for *M. chamomilla*; α-copaene (13.41 %), guaiol (9.35 %), safrole (8.35 %) and δ-cadinene (7.08 %) for *M. peruiferum*; linalool (28.80 %) and linalyl acetate (60.08 %) for *S. sclarea*; guaiol (48.29 %) and 2-undecanone (19.49 %) for *B. sarmientoi*; ethyl phthalate (13.09 %) and methyl-8-pimaren-18-oate (41.82 %) for *F. galbaniflua;* and neral (37.18 %) and geranial (5.02 %) for *M. officinalis*. According to Andrade et al. [20], EO from *C. dinisii* fresh leaves is composed mainly by α-pinene (35.41 %), β-pinene (17.81 %), sabinene (12.01 %) and bicyclogermacrene (7.59 %). EO from *S. guianensis* fresh leaves contains β-myrcene (13.14 %), germacrene-D (8.68 %) and bicyclogermacrene (16.71 %).

**Table 2:** Chemical composition of selected essential oils

Content (%)										
IRc	IRl	Component	*Matricaria chamomilla*	*Myroxylon peruiferum*	*Salvia sclarea*	*Bulnesia sarmientoi*	*Ferula galbaniflua*	*Melissa officinalis*	*Siparuna guianensis* ^a^	*Cinnamodendron dinisiia* ^a^
930	932	α-pinene	–	–	–	–	–	–	1.83	35.41
958	963	sabinene	–	–	–	–	–	–	–	12.01
961	970	β-pinene	–	–	–	–	17.34	–	–	17.81
969	971	6-metil-5-hepten-2-one	–	–	–	–	–	3.88	–	–
975	0980	β-myrcene	–	–	1.23	–	–	–	13.14	1.46
1018	1024	1,8-cineole	–	–	–	8.71	3.71	–	–	4.37
1086	1092	linalool	–	–	28.80	2.67	–	–	–	–
1135	1130	α-terpineol	–	–	5.14	–	–	–	–	–
1231	1235	neral	–	–	–	–	–	37.18	–	–
1247	1252	linalyl acetate	–	–	60.08	2.64	–	–	–	–
1263	1264	geranial	–	–	–	–	–	52.02	–	–
1282	1285	safrole	–	8.35	–	–	–	–	–	–
1287	1293	2-undecanone	–	–	–	19.49	–	–	1.69	–
1374	1374	α-copaene	–	13.41	–	–	–	–	–	–
1428	1432	trans-α-bergamotene	–	3.48	–	–	–	–	–	–
1443	1439	(+)-aromadendrene	–	2.27	–	1.26	–	–	–	–
1451	1455	(E)-β-farnesene	52.73	–	–	–	–	–	–	–
1455	1458	allo-aromandrendene	–	5.24	–	–	–	–	–	–
1470	1474	y-gurjunene	–	5.29	–	–	–	–	–	–
1476	1482	germancrene-D	3.42	–	–	–	–	–	8.68	–
1478	1479	ar-curcumene	–	5.05	–	–	–	–	–	–
1481	1487	β-selinene	–	3.27	–	–	–	–	–	–
1488	1497	bicyclogermancrene	–	–	–	–	–	–	16.71	7.59
1504	1505	(E,E)-a-farnesene	10.34	–	–	–	–	–	–	–
1504	1507	β-bisabolene	–	2.09	–	–	–	–	–	–
1510	1511	δ-amorfene	–	6.59	–	–	–	–	–	–
1520	1522	δ-cadinene	–	7.08	–	–	–	–	1.04	0.14
1576	1577	(−)-spathulenol	–	–	–	5.79	–	–	4.16	1.88
1594	–	diethyl phthalate	–	–	–	–	13.09	–	–	–
1601	1600	Guaiol	–	9.35	–	48.29	–	–	–	–
1630	–	(−)-sinularene	–	5.81	–	–	–	–	–	–
1652	1649	β-eudesmol	–	2.10	–	–	–	–	–	–
1656	1656	bisabolol oxide B	12.09	–	–	–	–	–	–	–
1657	1658	t-cadinol	–	–	–	1,35	–	–	4.14	–
1685	1685	α-bisabolol	9.83	–	–	–	–	–	3.35	–
1732	–	camazulene	2.30	–	–	–	–	–	–	–
–	–	elixene	–	–	–	–	5.87	–	–	–
–	–	methyl 8 (14)-pimaren-18- oate	–	–	–	–	41.82	–	–	–
–	–	NI	–	–	–	–	9.39	–	–	–
		Total identified (%)	90.72	79.38	94.05	88.85	81.83	93.08	55.25	80.67

IRl literature retention rate [22], IRc retention ratio calculated by Kovats’ equation. a) Described by Andrade et al. [20]

## Discussion

According to the classification of cytotoxicity and antileishmanial activity for extracts and fractions derived from plants and natural products defined by Study Program and Disease Control [27], the evaluated EOs are classified as moderately toxic (100 < CC_50_ ≤ 1000 μg/mL), except the EO from *S. guainensis*, which was classified as toxic (10 < CC_50 ≤_ 100 μg/mL). Regarding the antileishmanial activity only EOs from *S. guianensis*, *C. dinisii*, *M. chamomilla*, *C. verbenaceae*, *B. sarmientoi*, *F. galbaniflua* and *M. officinalis* are considered moderately active (50 < IC_50_ ≤ 150 μg/mL). The others are considered not active.

Considering the chemical composition of the EO from *M. chamomilla*, (E)-β-farnesene and (E,E)-α-farnesene were found as the major compound group representing 73.07 % of the total composition. These results corroborate with those reported by Machado et al. [28] that found farnesene derivatives as the most representative constituents (22 %) and their bioassays using EO from *Lantana camara* revealed a significant leishmanicidal activity against *L. amazonensis* (IC_50_/72 h = 0.25 μg/mL), except for the cytotoxic activity, in which the authors obtained high values on Brine shrimp (CC_50_ 10 μg/mL). Subsequently, Gawde *et al.* [29] observed that the chemical composition of *M. chamomilla* was similar to the one found in our study (β-farnesene, α-bisabolol oxide B, chamazulene) but no leishmanicidal activity on *L. donavani* was observed.

Studies on the chemical composition and biological activity of *M. peruiferum* EO are scarce. The literature reports (E) and (Z)-nerolidol, α-bisabolol and (E, E)-farnesol as its major components [30] but those compounds were not identified in the present study. Santos *et al.* [18] reported high levels of α-copaene in EO from *Copaifera reticulata* as well as for EO from *M. peruiferum*. The last one showed growth inhibitory activity for *L. amazonensis* with IC_50_/72 h values of 5 μg/mL for promastigotes and low cytotoxicity on J774G8 macrophages.

Ghannadi and Amree [31] have already described the EO composition obtained from the fresh oleogum resin and latex of Iranian *F. galbaniflua (synonym F. gummosa)* and the main constituents of this monoterpene rich oil were β-pinene (58.8 %). Other studies also indicate β-pinene as the major compound from the fresh oleogum resin and latex of this same specie [32, 33], which corroborates our results. The presence of methyl 8-(14)-pimaren-18-ate, a diterpene esters hydrocarbons, has been reported on rosin, a solid form of resin obtained from pines and some other plants; and also in the Cretaceous resins from India and Myanmar [34, 35]. To our knowledge, there is no antileishmanial activity reports related to this EO to date.

Rodilla et al. [36] determined the chemical composition of EO from *B. sarmientoi*. In accordance with our work, they identified guaiol as its major component. Studies with EO from *Endlicheria bracteolata*, which has 72.12 % of guaiol in its composition, showed IC_50_ of 7.93 μg/mL for *L. amazonensis* and presented a CC_50_ of 15.14 μg/mL for J774.G8 macrophages [37]. The antileishmanial activity may be attributed to the presence of a hydroxyl group of alcohol characteristics in the guaiol, especially in the exocyclic portion of the molecule [36].

The presence of linalyl acetate and linalool as the major compounds in *S. sclarea* EO (total of 88.88 %) corroborate to the results presented by Pitarokili et al. [38] that evaluated the EO composition of *S. sclarea* originated from two localities in Greece, and by Kuźma et al. [39] that evaluated the EO composition from *S. sclarea* plants generated *in vitro*. On the other hand, antileishmanial activity of linalool-rich EO from leaves of *Croton cajucara* against *L. amazonensis* was previously evaluated by Rosa et al. [40], they were able to demonstrate morphological changes in *L. amazonensis* promastigotes when treated with 15 ng/mL of that EO. In this study the cell lysis was observed within 1 h, indicating that the antileishmanial activity observed is directly related to the presence of linalool, due to the existence of a hydroxyl group in the organic alcohol function.

As in our study, the presence of the isomers of citral, neral and geranial are constantly reported in the chemical composition of the EO from *M. officinalis* [41–43]. Regarding the antileishmanial activity, Mikus et al. [44] reported an IC_50_/72 h of 7 μg/mL for *L. major*, a CC_50_/72 h of 25.5 μg/mL in HL-60 cells and SI of 3.6, higher than those observed in our study. Another study has already showed that citral presents activity against *T. cruzi*, possibly by inducing cell membrane lysis with leakage of cytoplasm [45].

The EO from *C. dinisii* and *S. guianensis* showed weak inhibitory effect on the protozoan *T. cruzi* with values of IC_50_/24 h = 209.30 μg/mL and 282.93 mg/mL, respectively. These values are higher when compared to those obtained in the study for *L. amazonensis*, 54.05 and 48.55 μg/mL, respectively [25].

The mechanism of action by which EOs inhibits parasite growth is still not well known, but previous studies have suggested that structural and morphological changes are caused by drugs that inhibit ergosterol synthesis, or interact with the membrane ergosterol [19, 46]. Other studies indicated that the activity of essential oils on parasites is mainly due to terpene composition. Terpenes are responsible for the hydrophobic characteristic of EOs, thus allowing their diffusion through the parasite cell membrane, affecting intracellular metabolic pathways and organelles [47].

## Conclusion

*F. galbaniflua* EO is effective against *L. amazonensis* promastigotes forms and has low cytotoxic activity. Thus, it represents a strong candidate for future studies in order to comprehend its biological activity agaisnt *L. amazonensis*.

The promising results of this study offer prospects for further research, as the evaluation of the antileishmanial potential of the major compounds and the elucidation of their molecules may, in the future, contribute to the discovery of effective drugs derived from plants for the treatment of parasitic diseases.
